# The Body Organs and Their Reconstruction Power (Regeneration) From the Viewpoint of Iranian-Islamic Physicians

**DOI:** 10.5812/ircmj.15193

**Published:** 2014-03-05

**Authors:** Mohsen Bahrami, Reza Mastery Farahani, Esmail Nazem, Mohammad Kamalinejad, Mansoor Keshavarz, Alireza Nikbakht Nasrabadi, Mohammad Yousofpoor, Farshad Amini Behbahani

**Affiliations:** 1 School of Traditional Medicine, Tehran University of Medical Sciences, Tehran, IR Iran; 2 Department of anatomy, School of Medicine, Shahid Beheshti University of medical Sciences, Tehran, IR Iran; 3 Department of Pharmacognosy, School of Pharmacy, Shahid Beheshti University of Medical Sciences, Tehran, IR Iran; 4 Department of physiology, School of Medicine, Tehran University of Medical Sciences, Tehran, IR Iran; 5 Tehran School of Nursing and Midwifery, Tehran University of Medical Sciences, Tehran, IR Iran; 6 Traditional and Complementary Medicine Research Center, Mazandaran University of Medical Sciences, Sari, IR Iran

**Keywords:** Physicians, Iran, Body, Organs

Dear Editor,

The purpose of the authors of this letter is to give a clear definition and classification of body parts from the viewpoint of Muslim and Iranian physicians during the Islamic Civilization era and to examine the views of Muslim scholars such as Ahvazi, Avicenna, and Amoli on the issue of the reconstruction power (regeneration) of organs. We aim to extract and bring into practice any useful and valuable points that can be found in their writings. Avicenna has discussed human body organs in the framework of the seven natural principles; elements, temperaments, humors, organs, vital spirits, faculties of power, and functions (1). The formation of the organs from the four humors takes place throughout the digestion steps in the body. Body organs are divided into two categories; simple organs and compound organs (2). In Avicenna’s view, simple organs are those organs whose constituting parts are of the same type, like bones and nerves (2, 3). Compound organs, on the other hand, are made up of several simple tissues, like hands and eyes (2, 3). Simple organs are either formed of semen (seminal) or formed of blood (sanguine) (2). Seminal organs are formed of the semen of the parents and the sanguine organs are formed of the blood of the mother and the child. For the latter group of organs, the semen of the mother and the menstrual blood are also to some extent responsible in forming the organs in the embryo (2, 3). Avicenna believes that every organ can be renewed as long as it is not very far from the fetal period, as in early years of childhood, because the power of the semen is still present in it (2, 3). Ageing changes the situation and reduces the reconstruction power. In sanguine organs, however, in case of abrasion or puncture, the organs are able to reconstruct again (2, 3). Today, the reconstruction power of most of the body organs of children, especially in the umbilical cord of newborns has been confirmed (2, 3). Another difference between seminal and sanguine tissues after birth is that seminal tissues cannot be repaired after injury unless these three conditions apply in addition juvenility:

1. The organ must not be a movable organ.
2. The organ must not be a sensitive organ.
3. The organ must not be in very quick blood circulation

If these conditions are not realized, the organ will be repaired with more delay (2-4). Seminal organs are describes as consisting of three subgroups: bones, nerves and vessels. Sanguine organs are divided into two subgroups: flesh and fat tissue (adipose) (2-6). Under the bones subgroup, different kinds of bones as well as cartilages and joints are discussed (2-6). The nerves subgroup includes nerves, ligaments, tendons, and membranes. In the vessels subgroup, veins, arteries, thick vessels and some nameless vessels are discussed (2-6). In the sanguine organs group, the flesh subgroup includes different kinds of flesh, muscles and glandular tissues and the subgroup contains fat tissues. These tissues together form the whole body and its compound organs such as the bones, the ribs, the heart and the liver (2-6). According to the experience of these physicians, most of the diseases which become chronic and difficult to cure happen during old age (7, 8). They related this to the decrease of natural heat and moist in seminal organs (7, 8). Both natural heat and natural moist are affected by circumstances such as mental stresses, food and medicines. Nowadays, some of these are taken into consideration for enhancing the immune system and organ renewal processes (7, 8). For example today *Aloe vera* could inhibit the anti-neoplastic immunitary response in patients treated with Phyto-therapy as major medicine use in Iranian traditional medicine (8, 9). The above reports reminds us of the situation of stem cells especially during the development period, since it has been shown today that stem cells are almost always present in different tissues of the body in different ages but they decline in quality and quantity as the person ages and various diseases harm the body. This is probably what Avicenna has considered the natural heat and moist of the body. With regard to the concentration of traditional physicians on the natural heat and natural moisture as the sources of regeneration especially in seminal organs, we suggest the development of culture environments using the medicines utilized by traditional physicians, in order to reduce the long-term effects of chronic and refractory diseases such as type 1 diabetes, blood cell malignancies, and diseases dealing with the regeneration of nerves, muscles, vessels and bones and will be very beneficial in future researches (Table 1).

**Table 1.** Organ Regeneration and Their Reconstruction Power From the Viewpoint of Iranian-Islamic Physicians

Organ	Sub Group
**Seminal Organs**	
Bones	bones, cartilages and joints
Nerves	nerves, ligaments, tendons and membranes
Vessels	arteries, veins and some nameless and thick (lymph) vessels
**Sanguine Organs**	
Flesh	flesh and muscles, glandular tissues
Adipose	fat tissues
